# Evaluation of the Possible Utilization of ^68^Ga-DOTATOC in Diagnosis of Adenocarcinoma Breast Cancer

**DOI:** 10.22038/aojnmb.2017.23695.1168

**Published:** 2018

**Authors:** Samaneh Zolghadri, Mojdeh Naderi, Hassan Yousefnia, Behrouz Alirezapour, Davood Beiki

**Affiliations:** 1 Material and Nuclear Fuel Research School, Nuclear Science and Technology Research Institute (NSTRI), Tehran, Iran; 2 Department of Chemistry, University of Zanjan, Zanjan, Iran; 3 Research Center for Nuclear Medicine, Shariati Hospital, Tehran University of Medical Sciences, Tehran, Iran

**Keywords:** Adenocarcinoma breast cancer, Gallium-68, PET/CT imaging

## Abstract

**Objective(s):**

Studies have indicated advantageous properties of [DOTA-DPhe^1^, Tyr^3^] octreotide (DOTATOC) in tumor models and labeling with gallium. Breast cancer is the second leading cause of cancer mortality in women, and most of these cancers are often an adenocarcinoma. Due to the importance of target to non-target ratios in the efficacy of diagnosis, the pharmacokinetic of ^68^Ga-DOTATOC in an adenocarcinoma breast cancer animal model was studied in this research, and the optimized time for imaging was determined.

**Methods:**

^68^Ga was obtained from ^68^Ge/^68^Ga generator. The complex was prepared at optimized conditions. Radiochemical purity of the complex was checked using both HPLC and ITLC methods. Biodistribution of the complex was studied in BALB/c mice bearing adenocarcinoma breast cancer. Also, PET/CT imaging was performed up to 120 min post injection.

**Results:**

The complex was produced with radiochemical purity of greater than 98% and specific activity of about 40 GBq/mM at optimized conditions. Biodistribution of the complex was studied in BALB/c mice bearing adenocarcinoma breast cancer indicated fast blood clearance and significant uptake in the tumor. Significant tumor: blood and tumor:muscle uptake ratios were observed even at early times post-injection. PET/CT images were also confirmed the considerable accumulation of the tracer in the tumor.

**Conclusion:**

Generally, the results proved the possible application of the radiolabelled complex for the detection of the adenocarcinoma breast cancer and according to the pharmacokenitic data, the suitable time for imaging was determined as at least 30 min after injection.

## Introduction

Radiopharmaceuticals are known as the powerful tools for early diagnosis of cancer and treatment of various malignancies. Subsequently, many radiolabelled compounds have been developed for diagnostic and therapeutic purposes in the recent years (1-2). Somatostatin receptors (SSTR) with high incidence on a wide range of human tumors such as the neuroendocrine, breast, lung, lymphomatic tissue and nervous system malignancies are the potential targets for peptide-receptor-targeted scintigraphy (3-4). Among the somatostatin analogs, octreotide and its derivatives with improved biological pharmacokinetics and targeting specificity have been widely used as imaging agents for somatostatin receptor scintigraphy (5-6).

^111^In-octreotide is the first and only Food and Drug Administration (FDA) approved SSTR-targeting radiopharmaceutical. While a series of radiolabeled octreotide analogs have been introduced, since then, [DOTA-DPhe^1^, Tyr^3^] octreotide (DOTATOC) has indicated advantageous properties in tumor models (6). Different radionuclides such as ^68^Ga, ^90^Y and ^177^Lu have been labeled with DOTATOC for diagnostic and therapeutic purposes (7-9).

Studies have shown that DOTATOC labeled with gallium is one of the best somatostatin analogs developed to date (10-11). This new imaging agent has demonstrated further sensitivity for the detection of neuroendocrine tumors (NETs) compared with the ^111^In-Octeroetide (12-13). However, the primary indication of ^68^Ga-DOTATOC peptide PET/CT is for the imaging of NETs with high expression of SSTRs, this radiolabeled complex can also be considered for the imaging of the other tumors with SSTRs as well as breast cancer (14).

According to the American cancer society statistics, about 1 in 8 (12%) women in the US will develop invasive breast cancer during their lifetime. Currently, breast cancer is the second leading cause of cancer death in women. Most breast cancers are often an adenocarcinoma, a type of carcinoma that starts in glandular tissue. Recent studies on the evaluation of FDG in the detection of breast cancer showed false-negative results in 12% cancer cases and as a result, some researchers believe that FDG PET should not be used for this purpose (15). Due to the important role of early detection in the response of treatment, development of new diagnostic methods is encouraging.

Lately, PET imaging of nude mice xenografted with the ZR-75-1 breast tumor cell line has been reported after injection of both ^68^Ga-DOTATOC and ^18^F-FDG. The results indicated the higher efficiency of ^68^Ga-DOTATOC in detecting breast tumors. The authors suggested the utilization of ^68^Ga-DOTATOC PET for the imaging of low-grade breast carcinomas not detected with ^18^F-FDG. In this research, the images were acquired only after 45 min of ^68^Ga-DOTATOC injection (16).

Deposit the importance of target to non-target ratio in the efficacy of diagnosis, to the best of our knowledge, no data has been reported representing the ratio and the pharmacokinetic of ^68^Ga-DOTATOC in an adenocarcinoma breast cancer animal model. In this study, ^68^Ga-DOTATOC was prepared and its biodistribution in the animal tumor models was studied at different intervals after injection by both scarification and PET/CT imaging.

## Methods

The ^68^Ge/^68^Ga generator (nominal activity of 50 mCi) was obtained from Pars Isotope (Karaj, Iran). DOTATOC was provided from ABX (Radeberg, Germany). All other chemical reagents were purchased from Sigma-Aldrich (Heidelberg, Germany). Whatman No. 2 paper was bought from Whatman (Buckinghamshire, U.K.). Radio-chromatography was performed using a thin layer chromatography scanner (Bioscan AR2000, Paris, France) and imaging studies were performed by means of a PET/CT scanner (Biograph 6 TrueX; Siemens Medical Solutions). The activity of the samples were measured by a p-type coaxial high-purity germanium (HPGe) detector (EGPC 80-200R) coupled with a multichannel analyzer card system (GC1020-7500SL, Canberra, U. S. A.). Calculations were based on the 511 keV peak. All values were expressed as mean ± standard deviation (Mean ±SD) and the data were compared using student T-test. Statistical significance was defined as P<0.05. Animal studies were performed in accordance with the United Kingdom Biological Council’s Guidelines on the Use of Living Animals in Scientific Investigations, second edition.

### Elution of ^68^Ge/^68^Ga generator

The appropriate eluent was selected after elution of the generator by 5 mL HCl with different concentrations from 0.1 to 1.0 M, while the activity of the eluted ^68^Ga was measured utilizing HPGe detector. In order to optimize the minimum required volume with the maximum radioactive concentration, the generator was eluted with 0.5 mL hydrochloric acid alternatively and each fraction was gathered in a separate vial. While, the activity of each fraction was measured by means of HPGe detector, the appropriate fractions for radiolabeling purposes were considered.

### Quality control of the eluted ^68^Ga

The radionuclidic purity of the eluted ^68^Ga from the generator was investigated by the determination of the decay pattern of ^68^Ga as well as gamma spectrometry of the decayed ^68^Ga samples. The decay pattern of ^68^Ga was determined following the half-life of the radionuclide by approximately 6 h. Also, for gamma spectrometry, the eluates were counted in an HPGe detector for 1,000 s after 48 h of the generator elution.

The content of the chemical impurities was determined by the inductively coupled plasma (ICP-OES) method. Radiochemical purity of the eluted ^68^Ga was studied using ITLC method and according to the reported procedure (17). For this purpose, ITLC chromatograms of ^68^GaCl3 solution in 10 % ammonium acetate:methanol on silicagel sheets and in 10 mM DTPA solution (pH∼4) on Whatman No. 2 paper were created.

### Radiolabeling of DOTATOC with ^68^GaCl_3_

A stock solution of DOTATOC with the concentration of 1 µg/µL in the distilled water was prepared. 16 µL of the stock solution was added to the vial containing 15 mCi of ^68^GaCl_3_ and the pH of the reaction mixture was adjusted to 4 utilizing HEPES. The reaction vial was put in the water bath with 90 °C for 10 min.

8 mL of water was then added to the final solution and the mixture was passed through a C_18_ Sep-Pak column preconditioned with 5 mL ethanol, 10 mL water and 10 mL air, respectively. The column was then washed with 1 mL ethanol and 9 mL of 0.9% NaCl.

### Quality control of the radiolabeled complex

Radiochemical purity of the radiolabeled complex was checked using both HPLC and ITLC methods. Paper chromatography was carried out using Whatman No. 2 paper and Acetonitrile:Water (1:1) as the mobile phase.

HPLC was performed on the final preparation, utilizing a C_18_ODS column with the dimensions of 100×4.6 mm and 5 µm particle size. Gradient elution was applied with the following parameters: A= water + 1% TFA, B= acetonitrile, flow rate: 2.6 mL min^-1^, 100 % A: 0 % B for 3 min, 50 % A: 50 % B for 7 min, 0 % A: 100 % B for 5 min.

### Developing breast adenocarcinoma-bearing BALB/c mice

A few BALB/c mice that previously injected with spontaneous Murine Mammary Adenocarcinoma Cells (MMAC) which has been histopathologically approved for invasive ductal carcinoma, grade II/III, (18) were provided from Pasteur Institute, Tehran, Iran. These mice breast tumor model were used for development of the tumor allograft in other healthy Balb/c mice.

For this purpose, the tumor allograft development was performed according to the previously reported procedure (19). Adenocarcinoma breast tumors of the mice were brought out and divided to 2–3 mm^3^ segments. These segments were implanted percutaneously in the right side of the flank of inbred female BALB/c mice (16–25 g, 6–8 weeks old, Pasteur Institute, Tehran, Iran). 24-31 days post implantation, the tumor volume reached 70–80 mm^3^.

### Biodistribution of the radiolabeled complex in BALB/c mice with adenocarcinoma breast cancer

100 μL of the radiolabeled complex with approximately 5.55 MBq radioactivity was injected intravenously into the BALB/c mice with adenocarcinoma breast cancer through their tail vein. The total amount of radioactivity injected into each animal was measured by counting the 1-mL syringe before and after injection in a dose calibrator with the fixed geometry. Biodistribution of the complex among tissues was determined by sacrificing the BALB/c mice at each selected time (15, 30 and 60 m) after injection using the animal care protocols.

Blood samples were rapidly taken after scarification. The tissues (heart, kidneys, spleen, stomach, intestine, lung, liver, skin, bone, muscle, adrenal, pancreas and tumor) were weighed and rinsed with normal saline and their activities were determined with a p-type coaxial HPGe detector coupled with a multichannel analyzer. The percentage of injected dose per gram (%ID/g) for different organs was calculated by dividing the activity amount of each tissue (A) to the decay-corrected injected activity and the mass of each organ.

### Imaging studies

5.55 MBq of final ^68^Ga-DOTATOC solution was injected intravenously into the mice through their tail veins. Static PET images were captured for 10 min with three sets of emission images starting 30, 60 and 120 min post injection. In addition, PET emission scans were preceded by CT scans performed for anatomical reference and attenuation correction (spatial resolution 1.25 mm, 80 kV, 150 mAs) with a total CT scanning time of 20 s. Reconstruction was performed using the iterative algorithm with attenuation correction. The reconstruction settings were 4 iterations and 21 subsets to a 256×256 matrix, with a post-filtering of 2 mm. Transmission data were reconstructed within a matrix of equal size by means of filtered back-projection, yielding a co-registered image set. The reconstructed emission images were reformatted into coronal, sagittal and maximum intensity projection (MIP) image sets.

Maximum, average and minimum standardized uptake values (SUV) for the observable organs in the images were calculated based on the region of interest (ROI) and according to Eq. 1:


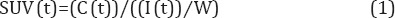


where C is the tissue radioactivity concentration (kBq/mL), I is the decay-corrected amount of injected radiolabeled complex [kBq], and w is the weight (g).

### Statistical analysis

Four rats were considered for each time interval. All values were expressed as mean ± standard deviation (Mean±SD) and the data were compared using Student’s T-test. P values of < 0.05 were considered statistically significant.

## Results

### Elution of ^68^Ge/^68^Ga generator

**Figure 1:**
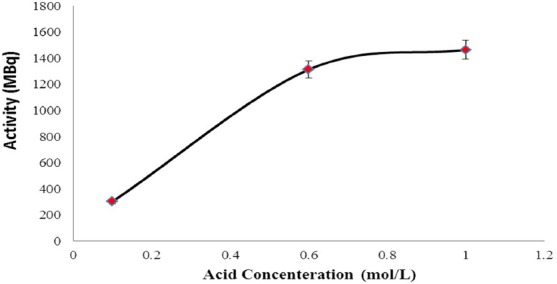
Activity of the eluted ^68^Ga (MBq) versus hydrochloric acid concenteration (mol/L) as the eluent

The generator was eluted by 5 mL HCl with different concentrations from 0.1 to 1.0 M. The results indicated the increment of the eluted ^68^Ga activity with the growth of hydrochloric acid concentration (Figure 1). Nevertheless, 0.6 M HCl was determined as the suitable solvent for the generator elution in the case of radiolabeling purposes.

Also, in order to optimize the procedure of elution, the generator was eluted with equal volumes of HCl, while the activity of each fraction was measured. Whereas only 0.5% of the total activity was observed in the first o.5 mL fraction, 90% of the whole ^68^Ga eluted activity was achieved in the four subsequent fractions.

With regard to the obtained results, the first fraction of the generator eluted by 0.5 mL of HCl was disregarded and the four next fractions (2.0 mL) were considered for radiolabeling purposes.

### Quality control of the eluted ^68^Ga

**Figure 2:**
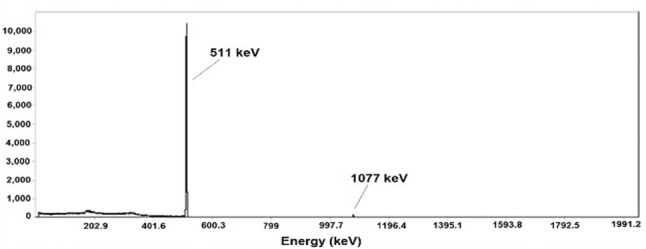
Gamma Spectrum of the eluted ^68^Ga after 48 h of the generator elution

**Figure 3:**
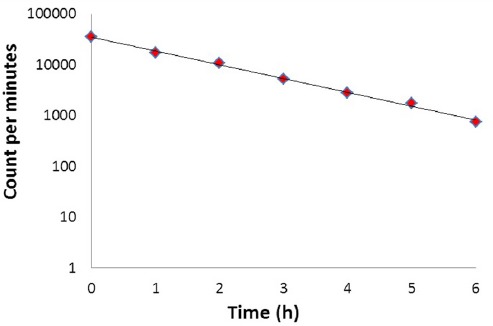
Activity of the eluted ^68^Ga (cpm) at different time after the generator elution (h)

The radionuclidic purity of the eluted ^68^Ga from the generator was investigated by the determination of the decay pattern of ^68^Ga and gamma spectrometry of the decayed ^68^Ga samples. The HPGe spectrum showed the presence of 511 and 1077 keV, all originating from ^68^Ga (Figure 2). The decay pattern of ^68^Ga was also studied up to 6 h (Figure 3). The half-life of ^68^Ga was calculated as 66.9±0.5 min which is about the reported half-life of ^68^Ga (67.629 min) (20).

The chemical purity of the eluate was checked after elution by both 0.6 and 1.0 M HCl. The average amount of metal ions in the eluted ^68^Ga is given in Table 1.

**Table 1:** The average amount of metal ions in the eluted ^68^Ga (ppm)

Eluent	tin	zinc	iron
0.6 M HCl	<0.1	0.230	0.380
1.0 M HCl	0.340	0.284	0.557

The radiochemical purity of the ^68^GaCl_3_ solution was checked in two solvent systems. In 10 mM DTPA solution, free ^68^Ga^3+^ is coordinated to a more lipophilic moiety as ^68^Ga(DTPA)^2-^ and migrates to a higher R_f_. On the other hand, in a 10 % ammonium acetate:methanol mixture (1:1), ^68^Ga^3+^ would remain in origin, while any other ionic cation of ^68^Ga^3+^ would migrate to higher R_f_ , which was not observed here.

### Preparation and Quality control of ^68^Ga-DOTATOC

**Figure 4:**
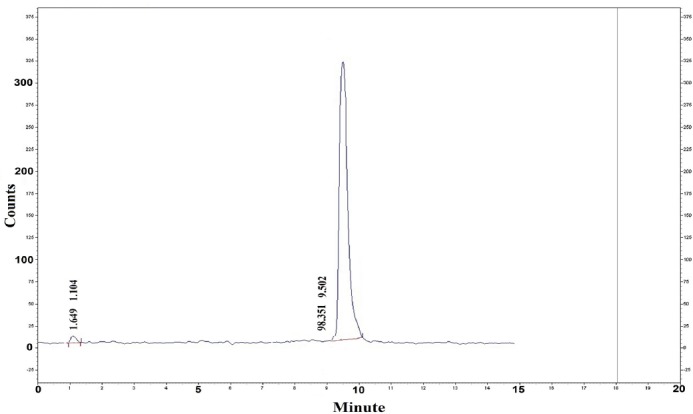
HPLC chromatogram of ^68^Ga-DOTATOC

The complex was produced with radiochemical purity of greater than 98% and specific activity of about 40 GBq/mM at optimized conditions. Radiochemical purity of the radiolabeled complex was checked using both HPLC and ITLC methods. HPLC analysis (Figure 4) showed that the fast eluting compound was hydrophilic ^68^GaCl_3_ cation (0.9 min), while ^68^Ga-DOTATOC with the heavy molecular weight was eluted after 8.98 min.

**Figure 5:**
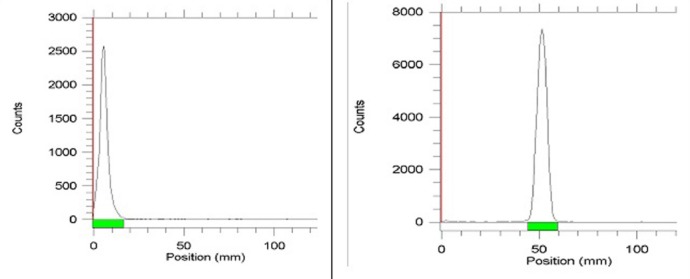
ITLC chromatogram of ^68^GaCl_3_ solution (left) and ^68^Ga-DOTATOC (right) in Acetonitrile:Water (1:1) using Whatman No. 2

In the case of ITLC (Figure 5), while free gallium cation remains in origin, ^68^Ga-DOTATOC elute faster as observed at R_f_. of 0.5. Both HPLC and ITLC studies approved the production of the radiolabeled complex with the radiochemical purity of greater than 98%.

### Biodistribution of the radiolabeled complex in BALB/c mice with adenocarcinoma breast cancer

**Figure 6:**
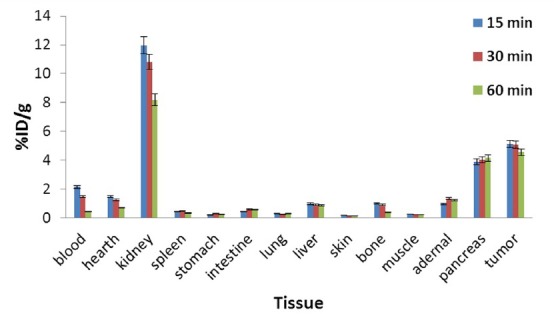
Percentage of injected dose per gram (%ID/g) at 15, 30 and 60 min after intravenously injection of ^68^Ga-DOTATOC (5.55 MBq) into the BALB/c mice with adenocarcinoma breast cancer

The tissue uptakes of ^68^Ga-DOTATOC were calculated as the percentage of the area under the curve of the related photo peak per gram of tissue (% ID/g) after intravenous administration of the radiolabeled complex into the BALB/c mice with adenocarcinoma breast cancer. Figure 6 shows the biodistribution of ^68^Ga-DOTATOC complex in breast adenocarcinoma model up to 60 min post injection.

### Imaging studies

**Figure 7:**
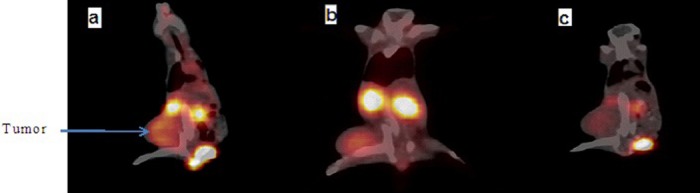
PET/CT fused images at 30 (a), 60 (b) and 120 (c) min after injection of ^68^Ga-DOTATOC (5.55 MBq) in the BALB/c mice with adenocarcinoma breast cancer

PET/CT images were captured at 30, 60 and 120 min after injection of ^68^Ga-DOTATOC in adenocarcinoma breast cancer (Figure 7). Also, maximum, average and minimum pixel SUVs of tumor were calculated. The mean amounts of SUV for tumor at the time of imaging were given in Table 2.

**Table 2:** The mean amounts of tumor SUV after injection of ^68^Ga-DOTATOC in the BALB/c mice with adenocarcinoma breast cancer

Time (min)	SUV
30	0.66
60	0.28
120	0.18

## Discussion

The elution of ^68^Ge/^68^Ga generator and quality control of the eluted ^68^Ga as well as determination of chemical ionic impurities shows the possibility of the ^68^Ga-radiolabeling using this generator to reach the desired radiochemical purity of the complex and the quality of the formulation for human applications (21).

However, with respect to the elution profile of ^68^Ge/^68^Ga generator (Figure 1), elution of the generator with 1.0 M HCl leads to higher activity of the ^68^Ga, but according to Table 1 and due to the higher amount of metal ions in the eluate, 0.6 M HCl was determined as the suitable solvent for the generator elution.

HPLC experiments on the radiolabeled complex using a gradient of water acetonitrile (0–100 244 100–0% added: 1% TFA) led to the fast removal of any free cation from the column with retention time (1.1 min), while ^68^Ga-DOTATOC with high molecular weight was eluted after 9.5 min (Figure 4).

Since the presence of metal cation impurities in ^68^Ga might interfere in the complexation of ^68^Ga with ligand, the concentrations of these metal ions in the ^68^Ga solution were determined using ICP-OES analysis. According to the results, the amount of zinc was 0.230 ppm in 2ml of ^68^GaCl_3_ solution with the activity of 1.3 GBq which is equal to 0.35 µg/GBq. While the acceptable limit of zinc and iron for ^68^Ge/^68^Ga eluate defined in the European Pharmacopoeia monograph is 10 µg/MBq (22), the results showed zinc and iron concentrations of much lower than this maximum extent. Also, in comparison to the other generators used for radiolabelling of DOTA-peptides (23-25) the amounts of chemical impurities for the in-house generator are comparable or fewer.

The biodistribution of ^68^Ga-DOTATOC demonstrated fast blood clearance and significant uptake in tumor and also in the somatostatin receptor-positive organs such as the pancreas. Rapid tumor uptake is observed with the maximum amount in 15 min post injection. While tumor uptake decrease slightly with time, the target: non target ratios for the radiolabeled complex in tumor bearing model at all intervals suggest that high quality images could be captured even at early times after injection. The tumor to muscle and tumor to blood activity ratios reach to the maximum amounts in 30 and 60 min post injection, respectively (Table 3).

**Table 3:** Tumor/blood and tumor/muscle ratios for ^68^Ga-DOTATOC in the BALB/c mice with adenocarcinoma breast cancer

Time (min)	Tumor/blood	Tumor/muscle
15	2.38	18.74
30	3.37	20.75
60	9.92	19.82

Significant radioactivity was also detected in the pancreas (maximum of 4.14) which is approximately constant with time. The kidney has the highest tissue radioactivity uptake and can be considered as a major body part of excretion. This result is consistent with the other DOTATOC radiolabeled complexes such as ^90^Y-DOTATOC where the kidney has been introduced as the dose limiting organ (26-27). However, in this case, considerable reduction in the kidney uptake with time as well as the short half-life and diagnostic nature of the ^68^Ga will significantly reduce the delivered absorbed dose in the kidneys. No significant accumulation is observed in the other organs^.^

The results of PET/CT images after injection of ^68^Ga-DOTATOC in adenocarcinoma breast cancer demonstrated that the only visible organs were the kidney, bladder and tumor. Other organs show slightly higher than the background activity and no lungs, liver and spleen uptake can be observed.

Although ^68^Ga-DOTATOC is often used for PET imaging of neuroendocrine tumors (28), some studies have pointed to somatostatin expression in breast carcinomas. However, further studies are needed to evaluate whether DOTATOC has diagnostic and/or potential therapeutic value in these tumor models. This study really shows the possible application of this agent for PET imaging of some breast carcinomas. The appropriate time of imaging studies with regard to target to non-target uptake ratios was determined as at least 30 min post injection.

## Conclusion

In this piece of research work, ^68^Ga-DOTATOC complex was prepared only after 10 min with radiochemical purity of greater than 98% at the optimized conditions. Biodistribution data was studied after intravenous injection of the complex to the BALB/c mice showing fast blood clearance and significant uptake in tumor as well as the other somatostatin receptor-positive organs. PET/CT images were acquired until 120 min post injection indicating considerable accumulation in the tumor. Biodistribution data showed that the maximum amounts for tumor to blood and tumor to muscle uptake ratio are 9.92 and 20.75 which occur in 60 and 30 min post injection, respectively. These results as well as the PET/CT results demonstrated the possibility of breast tumor imaging even at early time after injection of ^68^Ga-DOTATOC complex. Generally, it seems that this complex can be considered as an appropriate agent for the detection of the adenocarcinoma breast cancer especially in the patients with neuroendocrine tumors when they are candidates for PET imaging using ^68^Ga-DOTATOC. According to the target to non-target ratio, the appropriate time of imaging was at least 30 min post injection.
